# Alterations in the Multivariate Organization of Plasma Fatty Acid Profiles and Spontaneous Behavior in an AlCl_3_-Induced Rat Model of Neurotoxicity

**DOI:** 10.3390/biology15141162

**Published:** 2026-07-16

**Authors:** Muhammed Alzweiri, Ahmed S. A. Ali Agha, Nidal A. Qinna, Ghayda’ AlDabet, Heba Salah Abushahla, Thaqif El Khassawna, Talal Aburjai

**Affiliations:** 1 Department of Pharmaceutical Sciences, School of Pharmacy, The University of Jordan, Amman 11942, Jordan; hbh9220302@ju.edu.jo (H.S.A.); thaqif.elkhassawna@chiru.med.uni-giessen.de (T.E.K.); aburjai@ju.edu.jo (T.A.); 2 Department of Pharmacy, Pharmacological and Diagnostic Research Centre, Al-Ahliyya Amman University, Amman 19111, Jordan; asat3u@gmail.com; 3 Faculty of Pharmacy and Medical Sciences, University of Petra, Amman 11196, Jordan; nqinna@uop.edu.jo; 4 University of Petra Pharmaceutical Center (UPPC), Amman 11196, Jordan; ghayda.aldabet@uop.edu.jo; 5 Experimental Trauma Surgery, Faculty of Medicine, Justus-Liebig-University of Giessen, 35392 Giessen, Germany

**Keywords:** neurotoxicity, fatty acid profiling, behavioral organization, chemometrics, aluminum chloride

## Abstract

Current experimental neurotoxicity research commonly relies on evaluating individual behavioral and biochemical endpoints, often without considering whether spontaneous behavior and lipid metabolism exhibit coordinated multivariate organization under toxicological stress. To address this question, the present study employed an integrative framework combining spontaneous behavioral profiling, targeted plasma fatty acid methyl ester (FAME) profiling, chemometric analyses, targeted assessment of cholinergic function, and permutation-based statistical validation in an AlCl_3_-induced rat model of neurotoxicity. Behavioral assessment was performed using the Y-maze, and multivariate organization was evaluated using covariance analyses, principal component analysis (PCA), partial least squares discriminant analysis (PLS-DA), one-dimensional projection modeling, and nonparametric permutation testing (10,000 permutations). AlCl_3_ exposure was associated with early dose-related behavioral impairment at day 21 (ANOVA, *p* = 0.0006). In addition, both the measured fatty acid profiles and behavioral measures exhibited significant non-random multivariate organization, with progressive dose-related changes observed following AlCl_3_ exposure. Collectively, these findings suggest that neurotoxic exposure is associated with coordinated changes in the multivariate organization of measured fatty acid profiles, behavioral measures, and cholinergic function rather than isolated alterations in individual variables. Although confirmation in larger independent cohorts and broader molecular profiling remains warranted, the present findings support the use of multivariate organizational analysis as a complementary systems-level framework for investigating experimental neurotoxicity.

## Simple Summary

Neurotoxicity is commonly investigated by examining individual behavioral changes or specific biological markers. However, living systems operate through coordinated interactions among multiple biological components, and it remains unclear how these relationships change during toxic stress. In this study, we investigated whether exposure to aluminum chloride, a widely used experimental model of neurotoxicity, is associated with changes in the multivariate organization of spontaneous behavior and circulating fatty acid profiles. Rats exposed to increasing doses of aluminum chloride exhibited early behavioral impairment that became more pronounced with increasing exposure. More importantly, both behavioral measures and measured circulating fatty acid profiles exhibited significant non-random multivariate organization, with AlCl_3_ exposure being associated with progressive dose-related changes in these organizational patterns rather than random disruption. These findings suggest that neurotoxic responses may involve coordinated multivariate changes across multiple biological systems rather than isolated abnormalities. Understanding disease-related alterations at the level of multivariate biological organization may provide a complementary framework for investigating neurotoxicity and monitoring disease progression.

## 1. Introduction

Neurotoxicity is traditionally investigated through alterations in individual behavioral, biochemical, and molecular endpoints [1,2]. However, complex pathological responses often arise from interactions among multiple biological processes [3], and systems-level and network-medicine perspectives increasingly support the investigation of disease-associated changes in terms of coordinated network organization in addition to isolated abnormalities [4,5,6].

Lipids represent an important component of biological networks. Beyond their structural and energetic functions, lipids participate in cellular signaling and metabolic regulation, while lipid species are intrinsically interconnected through shared biosynthetic and remodeling pathways [7]. Consequently, lipidomics is increasingly used as a systems-level approach for characterizing lipid species, their biochemical interrelationships, and their roles in physiology and disease [8,9,10]. Moreover, because lipidomic datasets are compositional and statistically interdependent, relevant biological information is conveyed not only by individual component abundances but also by the multivariate covariance structure of the dataset, making appropriate compositional data analysis essential for biological interpretation [11,12,13,14].

Behavior likewise represents an integrative manifestation of organismal function. Complex behavioral phenotypes emerge from coordinated interactions among multiple neural systems and are increasingly being studied using phenomics and multivariate approaches capable of revealing latent behavioral organization beyond individual endpoints [15,16,17,18,19]. Despite extensive evidence showing that neurotoxic insults affect both metabolism and behavior, most studies have focused primarily on individual markers or average group differences, leaving it unclear whether toxicological exposure is associated with random disruption or coordinated changes in multivariate biological organization.

Aluminum chloride (AlCl_3_) is widely used as an experimental model of neurotoxicity because prolonged exposure induces cognitive impairment and biochemical alterations associated with neuronal dysfunction [20]. Although the AlCl_3_ model reproduces several key features of experimentally induced neurotoxicity, including cognitive and behavioral impairment and cholinergic alterations, it is best regarded as a mechanistic toxicological model rather than a comprehensive representation of human neurodegenerative disorders [20].

Within this context, the present study should be interpreted as an investigation of experimental AlCl_3_-induced neurotoxicity and is intended to characterize systems-level changes in the multivariate organization of measured plasma fatty acid profiles, spontaneous behavior, and cholinergic function under neurotoxic stress. Nevertheless, previous investigations have primarily focused on individual behavioral, biochemical, or molecular endpoints rather than systems-level biological organization.

Therefore, the present study employed an integrative framework combining spontaneous behavioral profiling, targeted plasma fatty acid analysis, chemometric modeling, and permutation-based validation to investigate whether chronic AlCl_3_ exposure is associated with changes in the multivariate organization of measured plasma fatty acid profiles and spontaneous behavioral measures. Rather than focusing exclusively on isolated variables, the study aimed to characterize neurotoxicity through the statistical organization of these measured biological variables and their cross-domain relationships.

## 2. Materials and Methods

### 2.1. Chemicals and Reagents

Methanol (HiPerSolv CHROMANORM, HPLC gradient grade, VWR Chemicals, Briare, France); potassium hydroxide pellets (≥85%, Analytical Reagent grade, AR); boron trifluoride in methanol (12% *w*/*w* BF_3_, 1.5 M, AcroSeal^®^, Thermo Scientific, Waltham, MA, USA); sulfuric acid (95–97%, J.T. Baker, Mallinckrodt Baker B.V., Deventer, The Netherlands); n-hexane (high-purity “PESTIPUR” grade, Carlo Erba Reagents S.A.S., Val-de-Reuil, France); iso-octane (GC grade, CAS 540-84-1; Bahadurgarh, Haryana, India); and dimethyl azelate (technical grade, 80%, Aldrich Chemistry, Sigma-Aldrich, St. Louis, MO, USA) used as the internal standard. A 37-component FAME reference mixture (FAME-37, Supelco, Bellefonte, PA, USA; Cat. No. 47885-U). Helium (≥99.999%, with O_2_/H_2_O traps) served as the carrier gas; nitrogen (≥99.999%) was used for evaporation.

### 2.2. Animals and Housing

Male Sprague–Dawley rats (average body weight ≈ 255 ± 10 g at the start of the experiment) were obtained and housed in the animal facility of the University of Petra (Amman, Jordan). Animals were acclimatized for at least 10 days prior to experimentation. Rats were maintained under controlled environmental conditions (temperature 22–24 °C, relative humidity 55–65%) on a 12 h light/12 h dark cycle, with ad libitum access to standard chow and water. Animals were group-housed (*n* = 8 per cage) under identical environmental conditions.

All procedures complied with institutional guidelines for the care and use of laboratory animals and with the principles of the Federation of European Laboratory Animal Science Associations (FELASA). The experimental protocol was approved by the local Institutional Animal Care and Use Committee at the University of Petra (IACUC Protocol Number: E/A/7/11/2025). Reporting followed ARRIVE guidelines; the completed checklist is provided as a separate file.

Given the established influence of gut microbiota on metabolic outcomes, microbiome-related variability was controlled during study design. A bedding-transfer procedure was implemented across cages during the acclimatization period to promote microbiome homogenization, following established normalization strategies (e.g., Miyoshi et al., 2018) [21]. In addition, all animals were maintained under identical dietary and environmental conditions and matched for strain, age, and sex.

**Figure 1:**
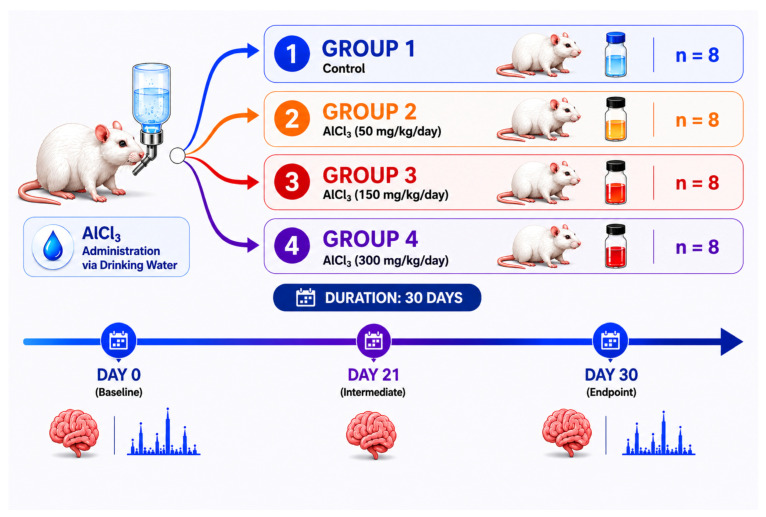
Experimental design and sampling schedule for the AlCl_3_-induced neurotoxicity model. Animals were assigned to four groups (*n* = 8 per group) receiving water (control) or AlCl_3_ at doses of 50, 150, or 300 mg/kg/day via drinking water for 30 days. Behavioral evaluation was performed on days 0, 21, and 30, while plasma fatty acid profiling was conducted at baseline (day 0) and endpoint (day 30).

The animals used in the present study represent a continuation of a previously characterized cohort, for which baseline (day 0) behavioral and fatty acids profiles have been reported (Ali Agha, 2026) [22]. Following baseline assessment, the same animals were maintained and randomly assigned to four experimental groups (*n* = 8 per group): a control group receiving drinking water only, and three treatment groups receiving aluminum chloride (AlCl_3_) dissolved in drinking water at doses of 50, 150, or 300 mg/kg/day for 30 consecutive days as illustrated in Figure 1. Daily drinking-water consumption was monitored on a per-cage basis throughout the treatment period. Average water consumption was approximately 35 mL/rat/day, with minimal between-group variation (estimated to be <5%) based on group-level bottle monitoring. Body weight was recorded for each animal at predefined intervals to verify the consistency of intended dose administration across experimental groups.

This longitudinal approach was consistent with the principles of reduction in animal use, enabling repeated assessment within the same cohort.

Behavioral assessments were conducted on days 21 and 30 of the treatment period using the Y-maze test. To minimize procedural variability, all animals were tested under identical conditions at both time points. At day 30, animals were subsequently subjected to blood collection for plasma fatty acid analysis using GC–MS.

### 2.3. Y-Maze Apparatus and General Behavioral Procedures

Locomotor activity, exploratory behavior, and self-grooming were assessed using a Y-shaped maze adapted from the classical spontaneous alternation paradigm, following previously established procedures (Ali Agha, 2026) [22]. The apparatus was constructed from wood and comprised three identical arms (75 cm × 15 cm) with 10 cm high sidewalls, positioned at 120° relative to each other to form a symmetrical Y-shaped configuration. The maze was located in a quiet room under stable and evenly distributed lighting conditions.

Behavioral testing was conducted at least 24 h after the final handling session to avoid transient stress-related effects. All experiments were carried out during the light phase within a fixed daily time interval to ensure consistency across animals.

At the start of each trial, rats were gently placed in the central junction of the maze, oriented toward the same arm. Each animal was then allowed to explore freely for 5 min. No food rewards or external stimuli were used, ensuring that behavior reflected spontaneous exploratory tendencies. To minimize potential olfactory interference, the apparatus was thoroughly cleaned and dried between trials.

### 2.4. Behavioral Scoring

Behavioral parameters were quantified by observers blinded to the fatty acid profiling outcomes. During each 5 min Y-maze session, arm entries were recorded as the total number of complete entries into each arm and were used as an index of locomotor activity. Spontaneous alternation behavior was additionally assessed as a measure of spatial working memory and was defined as consecutive entries into all three arms without repetition. The percentage of spontaneous alternation was calculated using the standard formula:

$$Alternation\;\left(\%\right)=\;\frac{Number\;of\;alternations}{Total\;arm\;entries\;-\;2}\;\times \;100$$

Investigatory behavior was evaluated by head-dip frequency, defined as instances in which the animal extended its head beyond the distal end or into the corners of an arm. Rearing activity was quantified as the number of vertical exploratory events in which the rat stood on its hindlimbs, with or without wall support.

Self-directed behavior was assessed by recording grooming activity, including both face-washing and body grooming, and expressed as the total number of discrete grooming bouts. Grooming latency was measured as the time (s) from placement in the maze center to the onset of the first grooming episode.

These measures were selected to capture complementary behavioral domains, with arm entries, head-dips, and rearing reflecting locomotor and exploratory activity, spontaneous alternation reflecting hippocampal-dependent working memory, and grooming-related parameters reflecting self-directed and affective-like behavior. The behavioral assessment framework was consistent with that previously described for baseline characterization of this cohort (Ali Agha, 2026) [22], enabling longitudinal comparison across time points.

Raw behavioral measurements for individual animals are provided in Supplementary Table S1 (Panels A–D) for day 21 and Supplementary Table S2 (Panels A–D) for day 30.

### 2.5. Blood Collection and Plasma Preparation

At the end of the 30-day treatment period, immediately following the final behavioral assessment, animals were anesthetized with isoflurane (induction ~5%, maintenance ~2.5% in 0.5 L/min oxygen) using a low-flow anesthesia system to minimize handling-induced stress. This approach was employed to reduce acute stress responses associated with blood sampling, which are known to rapidly alter circulating metabolite profiles [23]. Blood samples were then collected via the retro-orbital sinus using heparinized capillary tubes and transferred into EDTA-coated microtubes.

Samples were centrifuged at 6000× *g* for 10 min at 4 °C to separate plasma. The plasma fraction was carefully collected, aliquoted to avoid repeated freeze–thaw cycles, and stored at −80 °C until subsequent fatty acid profiling by GC–MS. All procedures were conducted in accordance with institutional ethical guidelines.

### 2.6. Plasma Fatty Acid Profiling by Orthogonal Dual-Column GC–MS

A targeted fatty acid methyl ester (FAME) profiling workflow was applied to plasma samples collected at the end of the treatment period from all experimental groups. This analytical approach, adapted from our previously described methodology (Ali Agha, 2026) [24], enables quantification of fatty acid-derived features rather than intact lipid classes, providing a chemically interpretable and pathway-relevant representation of systemic lipid metabolism. The method integrates an alkaline–acidic derivatization sequence with a dual-column GC–MS configuration employing two serially coupled capillary columns with orthogonal chromatographic selectivity, ensuring robust identification and quantification of fatty acid methyl esters (FAMEs).

**Figure 2:**
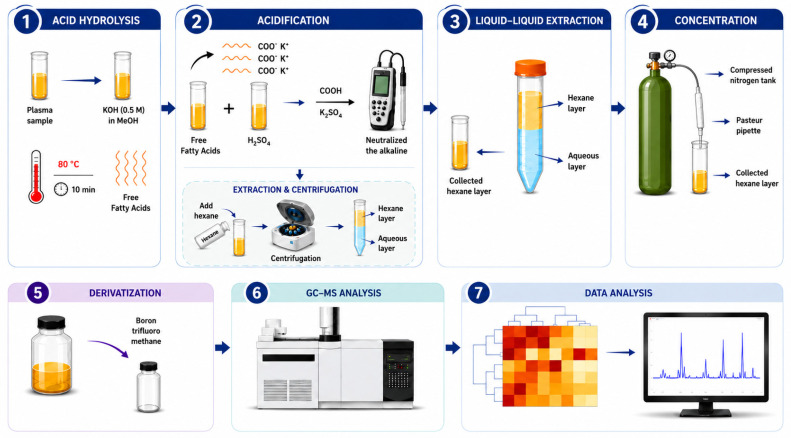
Schematic overview of the plasma free fatty acid analytical workflow. Plasma samples underwent alkaline hydrolysis, acidification, liquid–liquid extraction, nitrogen-assisted concentration, and derivatization with boron trifluoride–methanol prior to GC–MS analysis and downstream chemometric data processing.

Frozen plasma aliquots were thawed on ice and briefly vortexed. Lipids were saponified using 0.5 M KOH in methanol at 80 °C for 10 min to release fatty acids from esterified lipid species as shown in Figure 2.

Following cooling, concentrated sulfuric acid was added to neutralize the reaction. Fatty acids were extracted into n-hexane via liquid–liquid partitioning, and the organic phase was collected and evaporated to dryness under a gentle nitrogen stream to minimize oxidative degradation.

The dried extract was reconstituted in boron-trifluoride–methanol reagent (≈12% BF_3_, 1.5 M) to complete transmethylation. Resulting FAMEs were transferred to amber GC vials and analyzed immediately. Dimethyl azelate was added as an internal standard prior to injection to correct for injection variability. Each sample was analyzed once (technical replicate *n* = 1), with biological replication provided by independent animals within each experimental group. This approach was adopted because the plasma volume obtained from each animal was limited and prioritized for a single standardized GC–MS analysis. Additional blood collection or larger-volume sampling would have increased animal stress and potentially influenced circulating lipid profiles [23].

All samples were processed under identical experimental conditions, including standardized derivatization procedures and a consistent GC–MS configuration, with internal standard normalization applied to minimize technical variability.

#### 2.6.1. GC–MS Instrumentation

Analysis of FAMEs was performed using a GC–MS system configured with two capillary columns connected in series to achieve orthogonal separation. The primary column employed a polar polyethylene glycol stationary phase (TRB-Wax Ω, 20 m × 0.125 mm, 0.125 µm), coupled to a secondary low-polarity column (SCION-5MS, 30 m × 0.25 mm, 0.25 µm). Helium was used as the carrier gas at a constant flow of approximately 1.0 mL/min.

Sample aliquots (1 µL) were introduced via on-column injection, with the injector temperature maintained at 250 °C. Chromatographic separation was achieved using a temperature gradient starting at 100 °C (1 min hold), followed by a linear increase of 10 °C/min to 250 °C, and a final hold of approximately 40 min, resulting in a total run time of ~56 min. This program enabled effective resolution of fatty acids across a broad chain-length range.

Mass spectrometric detection was conducted under electron ionization (EI) conditions at 70 eV. Data acquisition was performed in centroid mode over an *m*/*z* range of 40–500. A solvent delay of approximately 6 min was applied to prevent interference from early-eluting solvent components.

#### 2.6.2. Identification and Selection of Lipid Variables

Identification of individual FAMEs was based on a combined evaluation of mass spectral data and chromatographic retention characteristics. Initial compound assignment was achieved through comparison with the NIST electron ionization (EI) mass spectral library, applying a minimum match threshold of 700.

To support retention-time reliability, all analytes were cross-referenced against a Supelco 37-component FAME standard analyzed under the same dual-column GC conditions. Given potential variability in retention behavior across column systems, the elution sequence was further evaluated by comparison with an external reference chromatogram obtained using an Omegawax 250 column (Supelco, Bellefonte, PA, USA) (courtesy of Prof. Luigi Mondello, University of Messina; Supelco reference material). Consistency in relative elution order across both systems provided an additional level of confirmation for chain length and degree of unsaturation.

### 2.7. Lipid Selection and Data Structure

Chromatographic processing, peak integration, and compound identification were performed as described previously [22]. Nine fatty acid species consistently detected across samples were selected for downstream analysis and designated Lipids 1–9. For each animal, lipid abundances were expressed as proportions relative to the total area under the curve of the selected lipid set, generating nine-dimensional compositional profiles.

Baseline (day 0) fatty acid profiling data for this cohort have been reported [22], and were used for longitudinal comparison. Endpoint (day 30) fatty acid profiles were generated in the present study following AlCl_3_ exposure. Due to technical losses during sample processing, the number of successfully analyzed samples varied slightly between time points. Baseline group sizes were *n* = 8, 7, 7, and 8, whereas day-30 group sizes were *n* = 7, 8, 8, and 8 for the control and increasing AlCl_3_ dose groups, respectively. No animal loss occurred during the experimental period.

Fatty acid profiling data were analyzed at the individual-animal level and, where appropriate, integrated with behavioral measures to evaluate treatment-associated changes in lipid composition and lipid–behavior relationships.

### 2.8. In Vivo Brain Protein Extraction and ELISA Analysis

Following completion of the experimental protocol at day 30, animals were sacrificed and brain tissues were rapidly excised, rinsed in ice-cold saline, and stored at −80 °C until analysis. Tissues were homogenized under cold conditions in an appropriate lysis buffer, and the homogenates were centrifuged to obtain clear supernatants for protein-based assays.

Total protein concentration in each sample was determined using the Bradford assay to ensure normalization across samples. Protein concentrations were calculated from a standard calibration curve, and all subsequent measurements were expressed relative to total protein content to minimize inter-sample variability.

Protein-level alterations associated with cholinergic function were quantified using commercially available sandwich enzyme-linked immunosorbent assay (ELISA) kits. Acetylcholinesterase (AChE) was measured in brain homogenates according to the manufacturer’s instructions.

Absorbance was recorded using a microplate reader at the specified wavelengths for each assay, and analyte concentrations were determined from standard calibration curves.

### 2.9. Statistical Analysis and Chemometric Modeling

Statistical and chemometric analyses were performed using complementary analytical frameworks implemented in Python (version 3.10), R (version 4.5.1), GraphPad Prism (version 8.4.3), and SIMCA-P (Version 14.1), enabling integrated evaluation of fatty acid profiling, behavioral, and biochemical datasets.

#### 2.9.1. Data Preprocessing and Normalization

Data handling and matrix construction were performed using pandas, with numerical operations implemented using NumPy v2.3.5. Lipid variables were expressed as relative abundances and treated as compositional data. Prior to multivariate analysis, zero values were replaced using half the minimum non-zero value for the corresponding lipid variable, followed by centered log-ratio (CLR) transformation to minimize compositional bias. All preprocessing steps were applied uniformly across samples to ensure consistency and reproducibility.

#### 2.9.2. Chemometric Modeling and Dimensionality Reduction

Chemometric analyses were conducted using Python (scikit-learn) and SIMCA-P software to systematically evaluate variation in fatty acid profiles across baseline, control, and AlCl_3_-induced conditions.

Principal Component Analysis (PCA) was initially applied as an unsupervised exploratory method to characterize the dominant sources of variance within the dataset without incorporating class labels. Two-dimensional PCA score plots (PC1–PC2) were examined to assess whether separation between healthy samples (baseline day 0 and day 30 control group) and AlCl_3_-induced groups (AD-B, AD-C, and AD-D) emerged as a primary driver of global variance. Class annotations were used solely for visualization and did not influence model construction.

To further interrogate treatment-associated structure, Partial Least Squares–Discriminant Analysis (PLS-DA) was subsequently employed as a supervised approach. A binary classification model was first constructed to distinguish healthy samples (class 0; baseline and control group) from AlCl_3_-induced samples (class 1; AD-B, AD-C, and AD-D). A multiclass model was then applied to assess dose-related effects, with healthy samples assigned to class 0 and AlCl_3_-induced groups assigned to classes 1 (AD-B), 2 (AD-C), and 3 (AD-D).

Given the observed overlap in both unsupervised and supervised projections, additional dimensionality assessment was performed using SIMCA-P through one-dimensional projection of principal component scores (1D PCA). In this step, all healthy samples (baseline and control group) were considered collectively and compared against AlCl_3_-induced groups (AD-B, AD-C, and AD-D) to evaluate the extent to which treatment-related variation could be resolved along dominant components.

Model performance was evaluated using cross-validation, with leave-one-out cross-validation for the healthy-versus-all-treated comparison and 7-fold cross-validation for pairwise comparisons. Complete model performance statistics, including R^2^X, R^2^Y, Q^2^, cross-validation procedures, and permutation testing (1000 permutations), are reported in Supplementary Table S4.

#### 2.9.3. Correlation and Integrative Analysis

Complementary statistical analyses were performed in R to characterize the multivariate organization of behavioral and fatty acid profiling systems and to evaluate how this organization evolved across experimental time points. Pearson correlation coefficients were computed to assess association structure within and between data domains, using CLR-transformed lipid variables where fatty acid profiling data were included.

At day 21, correlation analysis was confined to behavioral variables, with behavior–behavior matrices constructed to define the intrinsic organization of locomotor, exploratory, grooming, and spontaneous alternation measures at an early stage of AlCl_3_ exposure.

At day 30, correlation analyses were extended to incorporate both fatty acid profiling and behavioral datasets. Lipid–lipid and behavior–behavior correlation matrices were first generated independently to characterize domain-specific organization, followed by combined lipid–behavior correlation matrices to assess cross-domain relationships between plasma fatty acid profiles and behavioral systems.

Given the exploratory and systems-level focus of the study, interpretation emphasized overall covariance structure, effect size, and directionality rather than isolated statistically significant pairwise correlations. The Benjamini–Hochberg false discovery rate (FDR) correction was applied to control for multiple comparisons, and adjusted *p*-values are reported in the Supplementary Material.

To determine whether the observed fatty acid profiles and behavioral covariance structures exceeded random expectations, permutation-based null model analyses were performed using 10,000 randomized datasets. Lipid variables and behavioral variables were independently shuffled across animals while preserving the distribution of individual variables, thereby disrupting covariance relationships. For each permuted dataset, mean absolute correlation (MAC) and the proportion of variance explained by the first principal component (PC1) were recalculated and compared with the corresponding observed values. Permutation-derived *p*-values were calculated as the proportion of randomized datasets exhibiting values equal to or greater than those observed in the original datasets.

For the behavioral parameter “latency to first grooming,” animals that did not exhibit grooming during the 5 min observation period were assigned a value of 300 s to standardize the dataset. This adjustment did not alter the overall multivariate structure.

Biochemical endpoints derived from ELISAs were not incorporated into correlation analyses and were instead interpreted independently within the overall systems framework of the study.

#### 2.9.4. Analysis of ELISA and Behavioral Endpoint Data

ELISA-derived protein levels and behavioral endpoint variables, including spontaneous alternation percentages, were analyzed using GraphPad Prism (version 8.0.2). These analyses were conducted to evaluate group-level differences in biochemical and functional outcomes, complementing the multivariate chemometric framework.

Group comparisons were performed using one-way analysis of variance (ANOVA) followed by Tukey’s post hoc test.

Results are presented as mean ± standard deviation (SD), and statistical significance was defined as *p* < 0.05.

## 3. Results

### 3.1. Plasma Lipid Species Detected Across Cohorts

The plasma lipid variables analyzed in the present study correspond to the same nine fatty acid-derived species that were consistently detected, reliably quantified, and analytically validated in our previous baseline characterization study [22]. These include a panel of saturated, monounsaturated, and polyunsaturated fatty acids spanning the C13–C18 range, reflecting key components of systemic fatty acid metabolism.

Detailed compound identification, chromatographic resolution, and spectral confirmation have been reported previously and are not reiterated here. In the current work, these lipid species are treated as a fixed set of targeted fatty acid variables, enabling comparative analysis of their multivariate organization and covariance structure under AlCl_3_-induced neurotoxic conditions.

### 3.2. Changes in the Multivariate Organization of Plasma Fatty Acid Profiles Under AlCl_3_ Exposure

**Figure 3:**
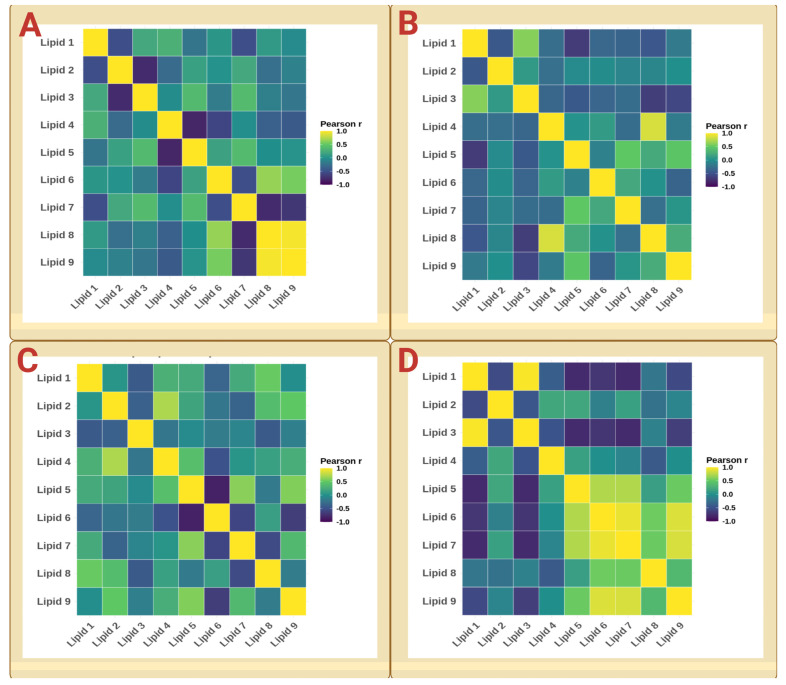
Plasma lipid correlation structure at day 30 under AlCl_3_ exposure. Heatmaps show Pearson correlation matrices of nine plasma lipid variables (Lipids 1–9) for (**A**) control (water), (**B**) 50 mg/kg/day, (**C**) 150 mg/kg/day, and (**D**) 300 mg/kg/day AlCl_3_-treated groups. Color scale represents correlation strength and direction.

Lipid–lipid correlation matrices for the day-30 control group and the three AlCl_3_-treated groups are presented in Figure 3.

The day-30 water-treated control group exhibited a positive correlation between Lipid 8 and Lipid 9 (r = 0.97, q = 0.008), together with additional moderate correlations among lipid variables. A positive Lipid 8–Lipid 9 correlation was also observed after exposure to 50 mg/kg/day AlCl_3_ (Group B) (r = 0.94, q = 0.033), accompanied by a negative correlation between Lipid 8 and Lipid 1 (r = −0.93, q = 0.033) and additional correlations involving Lipids 3 and 4. In the 150 mg/kg/day group (Group C), multiple moderate correlations were detected, including positive covariance between Lipid 5 and Lipid 9, negative covariance between Lipid 5 and Lipid 6, and additional associations involving Lipids 2 and 4. In the 300 mg/kg/day group (Group D), strong positive correlations were observed among Lipids 4, 6, 7, 8, and 9, while Lipids 1 and 3 formed a strongly correlated pair. Complete Pearson correlation matrices are provided in Supplementary Table S3 (Panels A–D).

### 3.3. Chemometric Characterization of Multivariate Organization in Measured Fatty Acid Profiles Under AlCl_3_ Exposure

To further characterize multivariate organization beyond correlation analysis, complementary unsupervised and supervised chemometric approaches were applied to the plasma fatty acid dataset.

#### 3.3.1. Global Fatty Acid Profiling Variance Structure Assessed by PCA

**Figure 4:**
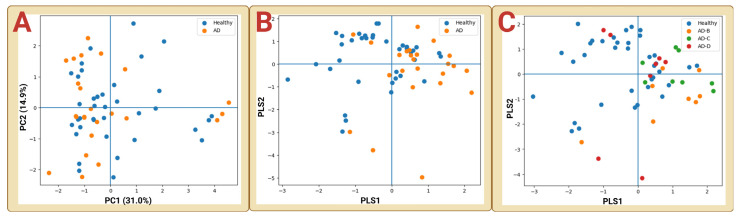
Two-dimensional chemometric representation of plasma fatty acid profiles under AlCl_3_ exposure. (**A**) PCA score plot (PC1–PC2) showing global variance structure with overlap between healthy and AlCl_3_-treated samples. (**B**) Binary PLS-DA score plot (PLS1–PLS2) illustrating partial separation between healthy and treated groups. (**C**) Multiclass PLS-DA score plot (PLS1–PLS2) showing partially overlapping group distributions across exposure groups.

Principal Component Analysis (PCA) was applied as an unsupervised method to evaluate the variance structure of fatty acid profiles. The first two principal components accounted for approximately 31% (PC1) and 15% (PC2) of the total variance (Figure 4A).

In the PC1–PC2 score plot, samples from the healthy, day-30 control, and AlCl_3_-treated groups exhibited overlapping distributions, with partial separation observed along PC1.

#### 3.3.2. Treatment-Associated Covariance Assessed by PLS-DA

Partial Least Squares–Discriminant Analysis (PLS-DA) was performed using both binary (healthy vs. AlCl_3_-treated) and multiclass (Healthy, AD-B, AD-C, AD-D) models.

Binary and multiclass PLS-DA score plots are shown in Figure 4B–C. Both models demonstrated partial group separation within the latent-variable space, although overlap among groups remained evident in the two-dimensional projections.

PLS-DA model performance was evaluated by cross-validation and permutation testing. Model performance statistics (R^2^X, R^2^Y, Q^2^, and permutation-test results) are presented in Supplementary Table S4.

Given the overlapping group distributions and the exploratory nature of the present dataset, the PLS-DA models were interpreted as descriptive visualizations of multivariate organization rather than predictive classification models.

#### 3.3.3. One-Dimensional Projection of Multivariate Organization in Measured Fatty Acid Profiles

**Figure 5:**
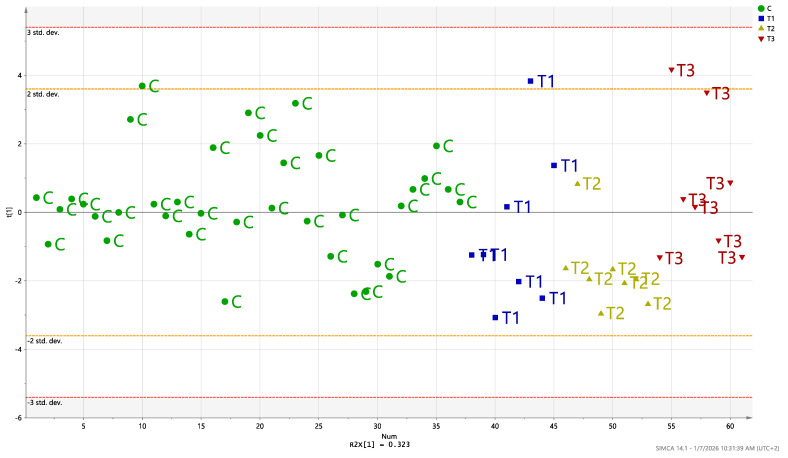
One-dimensional PCA projection of measured plasma fatty acid organizational patterns under AlCl_3_ exposure. Scores plot showing projection of samples onto the first principal component (t[1]). Healthy samples (C) form a distinct reference distribution, while AlCl_3_-treated groups (T1: 50 mg/kg/day, T2: 150 mg/kg/day, T3: 300 mg/kg/day) exhibit descriptive differences along the first principal component.

PCA scores were projected onto the first principal component (PC1) to examine the primary axis of variation (Figure 5).

Healthy samples (baseline and day-30 control) occupied one region of the first principal component, whereas the AlCl_3_-treated groups (AD-B, AD-C, and AD-D) were progressively distributed along the same axis according to treatment dose.

#### 3.3.4. CLR-Transformed PCA Validation of Fatty Acid Profiling Organization

**Figure 6:**
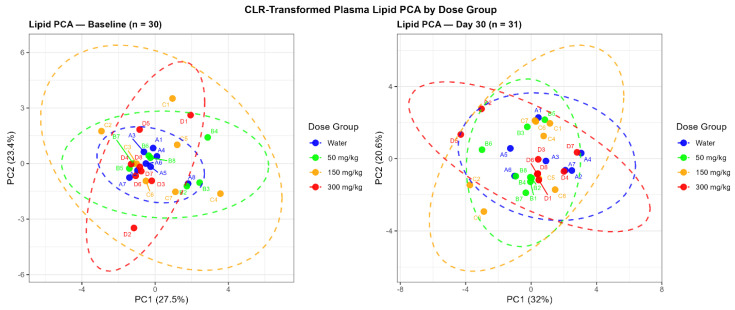
CLR-transformed PCA score plots of plasma fatty acid profiles at baseline and day 30. Two-dimensional PCA score distributions generated from CLR-transformed plasma fatty acid profiling data. Baseline (day 0) profiles showing group-specific dispersion patterns prior to exposure. Day 30 profiles following AlCl_3_ exposure, demonstrating altered multivariate organization across dose groups. Dashed ellipses represent group-level covariance distributions. PC1 and PC2 values indicate explained variance percentages.

Principal component analysis of CLR-transformed fatty acid datasets was performed at baseline (day 0) and day 30 (Figure 6).

At baseline, PCA demonstrated partially overlapping group distributions, with the water group exhibiting relatively constrained clustering, whereas the 50 and 150 mg/kg/day groups showed broader dispersion. Analysis of multivariate homogeneity of group dispersions also identified significant differences among baseline groups (*p* = 0.032). By day 30, redistribution of group positions in principal component space was observed, with PC1 explaining 32.0% of the total variance, compared with 27.5% at baseline.

#### 3.3.5. Permutation-Based Validation of Fatty Acid Profiling Covariance Structure

Permutation-based analyses were performed to evaluate covariance structure within the fatty acid profiling datasets (Table 1).

Permutation analysis identified an observed mean absolute correlation (MAC) of 0.233 among transformed lipid variables at baseline (day 0) (*p* < 0.0001), while the first principal component explained 27.5% of the total variance (*p* = 0.210). By day 30, the observed MAC was 0.211 (*p* < 0.001), and the first principal component explained 32.0% of the total variance (*p* = 0.023).

### 3.4. Changes in Spontaneous Alternation Performance Following AlCl_3_ Exposure

**Figure 7:**
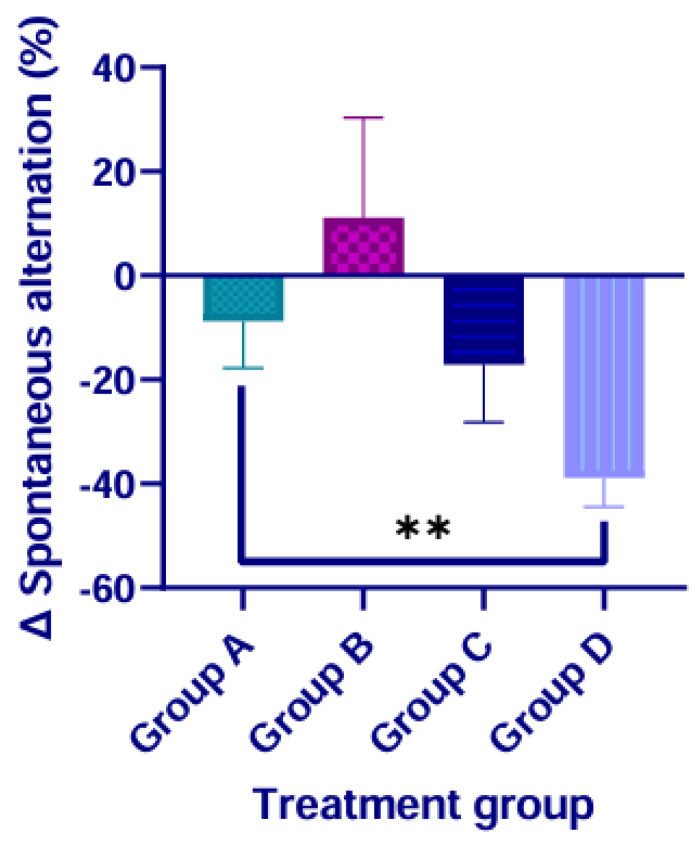
Change in spontaneous alternation at day 21 relative to baseline. Bar plot showing mean Δ spontaneous alternation (%) for Groups A–D at day 21 relative to day 0. Error bars represent standard deviation. ** *p* < 0.01.

Spontaneous alternation performance was evaluated relative to baseline (day 0) at both day 21 and day 30. Baseline spontaneous alternation values were comparable across all experimental groups (approximately 74–87%). Changes observed at day 21 are shown in Figure 7.

The control group (Group A) showed a mean change of approximately −8.8%. Group B (50 mg/kg/day) showed a mean change of approximately +11.1%, Group C (150 mg/kg/day) showed a mean reduction of approximately −16.9%, and Group D (300 mg/kg/day) showed a mean reduction of approximately −38.9%. Overall group differences were significant (ANOVA, *p* = 0.0006). Post hoc comparisons identified significant differences between Group D and Groups A and B and between Groups B and C.

**Figure 8:**
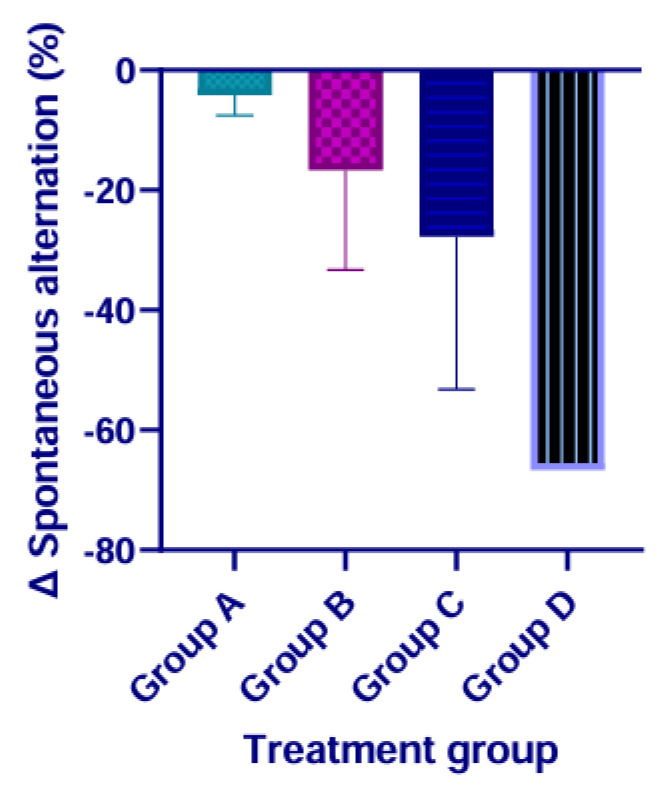
Change in spontaneous alternation at day 30 relative to baseline. Bar plot showing mean Δ spontaneous alternation (%) for Groups A–D at day 30 relative to day 0. Error bars represent standard deviation. Group D is represented by a single valid subject (*n* = 1) due to exclusion of animals not meeting exploration criteria, and is therefore shown for completeness without inference.

The corresponding changes at day 30 are presented in Figure 8.

At day 30, the control group showed a mean change of approximately −2.1%, whereas Groups B, C, and D showed mean reductions of approximately −15.6%, −27.8%, and −66.7%, respectively. Cross-sectional spontaneous alternation values ranged from approximately 64–67% in Groups A and B, approximately 50% in Group C, and approximately 33% in Group D. Overall statistical significance was not observed at day 30.

### 3.5. Changes in the Multivariate Organization of Behavioral Systems

Behavior–behavior correlation matrices were generated to examine the multivariate organization of behavioral variables at day 21 and day 30 following AlCl_3_ exposure. Complete Pearson correlation matrices with FDR-adjusted *q*-values are provided in Supplementary Tables S5 and S6.

#### 3.5.1. Behavioral Covariance at Day 21

**Figure 9:**
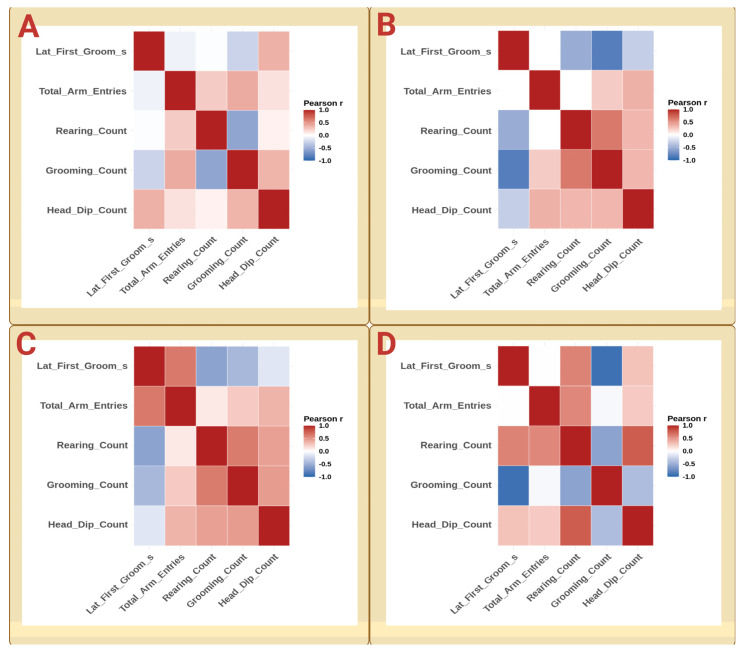
Behavioral correlation structure at day 21 across experimental groups. Heatmaps showing Pearson correlation matrices among Y-maze behavioral variables for Group A (panel (**A**)), Group B (panel (**B**)), Group C (panel (**C**)), and Group D (panel (**D**)) at day 21. Variables include latency to first grooming, total arm entries, rearing count, grooming count, and head-dip count.

Behavior–behavior correlation matrices obtained at day 21 are presented in Figure 9.

In the day-21 control group (Group A), weak-to-moderate correlations were present among exploratory and grooming-related variables. In the 50 mg/kg/day group (Group B), an inverse correlation between latency to first grooming and grooming frequency was identified, together with additional correlations involving grooming-related and exploratory variables. The 150 mg/kg/day group (Group C) displayed correlations among arm entries, head-dip behavior, rearing, grooming count, and grooming latency, although no single dominant correlation pattern was evident. In the 300 mg/kg/day group (Group D), a strong inverse correlation between latency to first grooming and grooming frequency remained significant after FDR correction. Positive correlations between rearing and exploratory variables were also identified.

#### 3.5.2. Behavioral Covariance at Day 30

**Figure 10:**
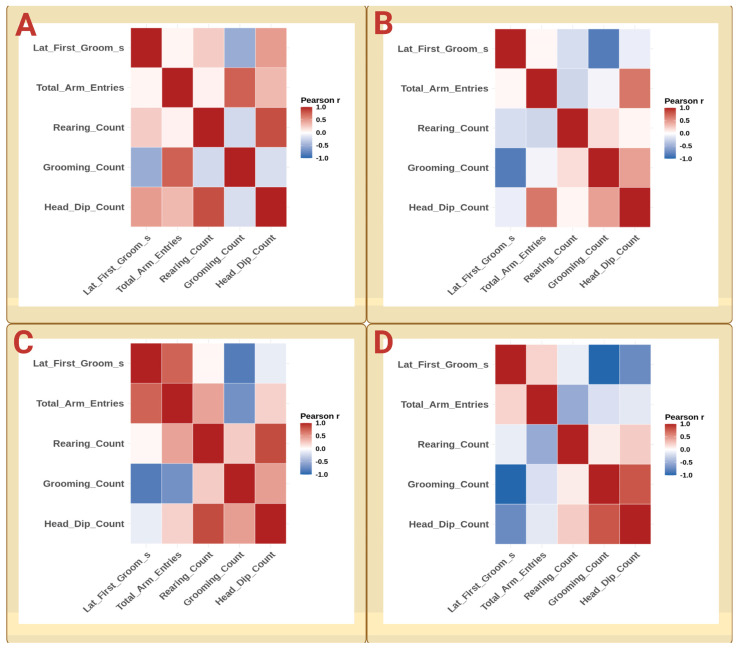
Behavioral correlation structure at day 30 across experimental groups. Heatmaps showing Pearson correlation matrices among Y-maze behavioral variables for Group A (panel (**A**)), Group B (panel (**B**)), Group C (panel (**C**)), and Group D (panel (**D**)) at day 30. Variables include latency to first grooming, total arm entries, rearing count, grooming count, and head-dip count. Across groups, behavioral relationships exhibit more consolidated and distinctly reweighted patterns compared to day 21.

Behavior–behavior correlation matrices obtained at day 30 are presented in Figure 10.

At day 30, the control group (Group A) showed a positive correlation between rearing and head-dip behavior together with a moderate inverse correlation between grooming frequency and grooming latency. An inverse correlation between grooming latency and grooming frequency was likewise detected in the 50 mg/kg/day group (Group B), where positive correlations were also observed among exploratory variables, including arm entries and head-dip behavior. At 150 mg/kg/day (Group C), this inverse correlation remained significant after FDR correction, together with a positive correlation between rearing and head-dip behavior. At the highest dose (300 mg/kg/day; Group D), the inverse correlation between grooming latency and grooming frequency also remained significant after FDR correction, while grooming frequency was positively correlated with head-dip behavior, and grooming latency was inversely correlated with head-dip count.

#### 3.5.3. PCA Validation of Behavioral Organization

**Figure 11:**
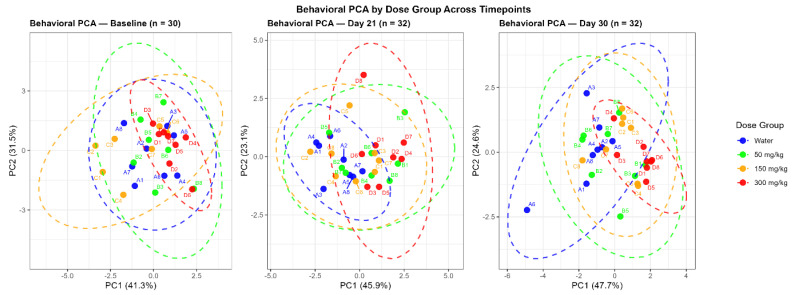
PCA score distributions of behavioral profiles across experimental groups and time points. Two-dimensional PCA score plots generated from centered and scaled behavioral variables at baseline (day 0), day 21, and day 30. Dashed ellipses represent group-level covariance distributions for each experimental group. PC1 and PC2 values indicate explained variance percentages.

Principal component analysis of centered and scaled behavioral datasets was performed for baseline (day 0), day 21, and day 30 (Figure 11).

At baseline (day 0), PC1 and PC2 explained 41.3% and 31.5% of the total variance, respectively. Exploratory behaviors (total arm entries, rearing count, and head-dip activity) were primarily associated with PC1, whereas grooming-related variables were primarily associated with PC2. At day 21, the proportion of variance explained by PC1 increased to 45.9%, while PC2 accounted for 23.1%. By day 30, PC1 explained 47.7% of the total variance, whereas PC2 accounted for 24.6%.

#### 3.5.4. Permutation-Based Validation of Behavioral Covariance Structure

Permutation-based analyses of behavioral covariance structure are summarized in Table 2.

At baseline (day 0), the observed mean absolute correlation (MAC) among behavioral variables was 0.262 (*p* = 0.002), and the first principal component explained 41.3% of the total variance (*p* = 0.002). The observed MAC increased to 0.313 at day 21 (*p* < 0.0001), with PC1 explaining 45.9% of the total variance (*p* < 0.0001). By day 30, the observed MAC reached 0.336 (*p* < 0.0001), while PC1 explained 47.7% of the total variance (*p* < 0.0001).

### 3.6. Changes in the Multivariate Organization of Fatty Acid–Behavior Coupling at Day 30

**Figure 12:**
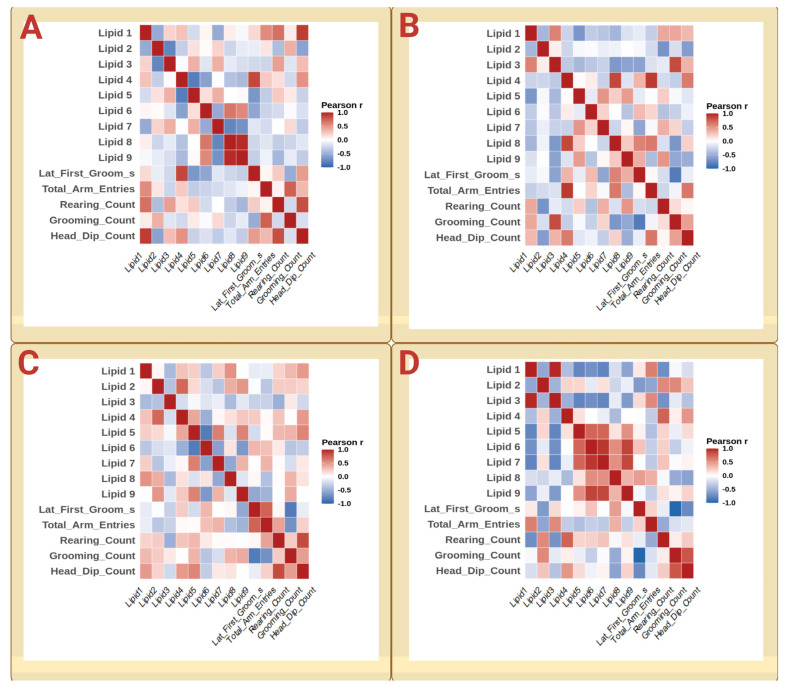
Integrated lipid–behavior correlation structure at day 30. Heatmaps show Pearson correlation matrices combining plasma lipid variables (Lipids 1–9) and Y-maze behavioral measures across groups at day 30. Panels correspond to Group A (control, panel (**A**)), Group B (50 mg/kg/daypanel (**B**)), Group C (150 mg/kg/day, panel (**C**)), and Group D (300 mg/kg/day, panel (**D**)). Color intensity represents the strength and direction of correlations (red, positive; blue, negative).

Integrated lipid–behavior correlation matrices at day 30 are presented in Figure 12. Complete Pearson correlation matrices with FDR-adjusted q-values are provided in Supplementary Table S7 (Panels A–D).

In the day-30 control group (Group A), positive correlations were observed between Lipid 1 and arm entries, rearing, and head-dip behavior. Lipids 5 and 6 were inversely correlated with grooming latency, whereas Lipid 4 was positively correlated with grooming latency and head-dip behavior. At 50 mg/kg/day (Group B), positive correlations between Lipids 4 and 8 and arm entries were accompanied by additional positive correlations with grooming latency and head-dip behavior. Lipid 3 was also positively correlated with grooming count, while Lipid 9 exhibited inverse correlations with arm entries, grooming count, and head-dip behavior together with a positive correlation with rearing. At 150 mg/kg/day (Group C), Lipids 1 and 5 were positively correlated with head-dip behavior, whereas Lipid 9 was inversely correlated with grooming latency and arm entries. Lipid 6, in turn, was positively correlated with grooming latency and negatively correlated with head-dip behavior. At 300 mg/kg/day (Group D), Lipid 2 exhibited an inverse correlation with grooming latency together with a positive correlation with grooming count. Lipid 8 was positively correlated with grooming latency and negatively correlated with grooming count and head-dip behavior. Positive correlations between Lipid 4 and both rearing and head-dip behavior were also observed, whereas Lipids 1 and 3 were positively correlated with arm entries and inversely correlated with rearing.

Group-specific descriptive lipid–behavior correlation patterns were observed; however, no individual associations remained significant after FDR correction. Accordingly, the matrices should not be interpreted as evidence of specific lipid–behavior relationships.

### 3.7. In Vivo Biochemical Endpoints Under AlCl_3_ Exposure

**Figure 13:**
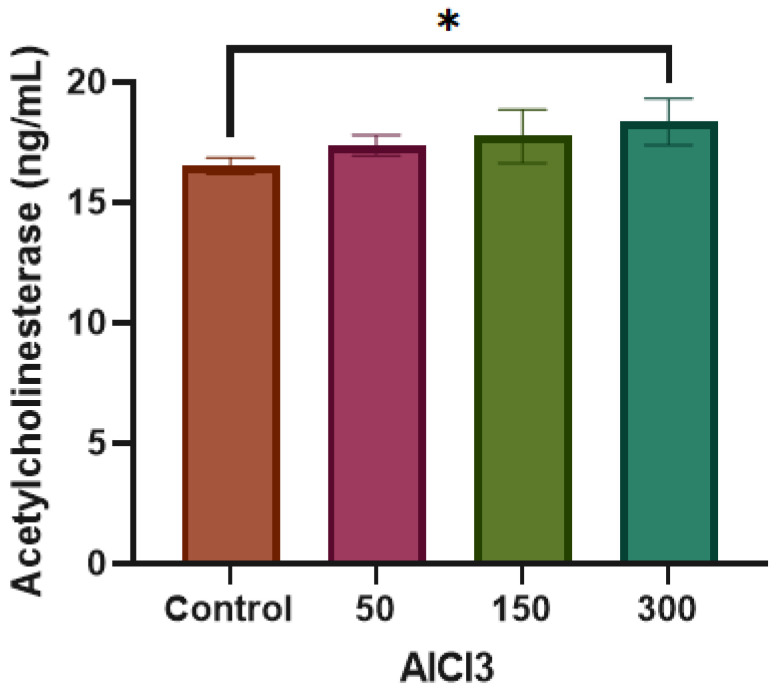
Effect of chronic AlCl_3_ exposure on brain acetylcholinesterase (AChE) levels. Bar plot showing AChE concentrations (ng/mL) in brain homogenates of rats receiving water (control) or AlCl_3_ at doses of 50, 150, or 300 mg/kg/day for 30 days. Data are presented as mean ± SD. A modest dose-related increase in AChE levels was observed, with a significant elevation in the 300 mg/kg/day group compared with the control group (*p* = 0.0159). * = *p* < 0.05.

Brain acetylcholinesterase (AChE) levels were measured in homogenates collected at day 30. The corresponding results are presented in Figure 13.

Mean AChE concentrations were 16.52 ng/mL in the control group and increased to 17.37, 17.75, and 18.36 ng/mL in the 50, 150, and 300 mg/kg/day treatment groups, respectively. Statistical analysis identified a significant increase in the 300 mg/kg/day group compared with the control group (*p* = 0.0159).

## 4. Discussion

### 4.1. Interpreting AlCl_3_-Induced Neurotoxicity from a Multivariate Organizational Perspective

The present study investigated whether chronic AlCl_3_ exposure was associated with alterations in the multivariate organization of targeted plasma fatty acid profiles and spontaneous behavioral measures rather than focusing exclusively on individual endpoints. Across the complementary analytical approaches employed, both the measured fatty acid and behavioral datasets exhibited structured covariance patterns that differed among the experimental groups. These organizational changes were accompanied by alterations in spontaneous alternation performance and a significant increase in brain AChE levels at the highest AlCl_3_ dose, whereas no individual lipid–behavior correlations remained significant after correction for multiple testing.

Collectively, these findings suggest that the response to chronic AlCl_3_ exposure is reflected not only in selected biochemical and behavioral measurements but also in the statistical organization of the measured variables. This observation is consistent with the concept that biological responses emerge from coordinated interactions among multiple components rather than from isolated variables alone [25,26,27]. Within the context of the present study, the observed covariance structures therefore provide complementary information to conventional univariate analyses and offer an additional perspective for characterizing experimental neurotoxicity.

At the same time, these findings should be interpreted within the scope of the experimental design. The analyses were based on a targeted panel of nine plasma fatty acid species and relatively modest group sizes, and therefore the observed organizational patterns should be regarded as exploratory statistical associations within the measured datasets rather than definitive evidence of systems-level biological remodeling or mechanistic state transitions.

### 4.2. Multivariate Organization of Measured Plasma Fatty Acid Profiles

The measured plasma fatty acid variables exhibited structured covariance patterns across all experimental groups, although the relationships among these variables changed following AlCl_3_ exposure. In the control group, the strongest association was observed between Lipids 8 and 9, whereas AlCl_3_-treated groups exhibited additional positive and negative associations involving several measured fatty acid variables. Collectively, these findings suggest that chronic AlCl_3_ exposure was associated with changes in the covariance structure of the measured plasma fatty acid profiles.

The complementary chemometric analyses further supported this interpretation. Although the PCA and PLS-DA score plots demonstrated only partial separation among the experimental groups, permutation analyses further supported the presence of non-random covariance organization within the measured fatty acid dataset at both baseline and day 30.

In addition, the proportion of variance explained by the first principal component increased from 27.5% at baseline to 32.0% following AlCl_3_ exposure, indicating that a greater proportion of the variability within the measured fatty acid dataset was captured along a dominant multivariate axis. Collectively, these findings suggest that chronic AlCl_3_ exposure was associated with changes in the statistical organization of the measured fatty acid profiles that complement conventional univariate analyses.

The overlap observed in the PCA and PLS-DA score plots should also be interpreted in the context of the dataset’s baseline characteristics. Significant differences in multivariate dispersion were already present before exposure, indicating that the experimental groups did not originate from completely identical multivariate backgrounds despite random allocation and standardized experimental conditions.

Consequently, part of the multivariate differences observed at day 30 may reflect baseline heterogeneity in addition to exposure-associated changes, and the findings should therefore be interpreted with appropriate caution.

This interpretation is further supported by the exploratory nature of the multivariate analyses and the absence of complete group separation within the low-dimensional chemometric projections.

From a biological perspective, these findings are consistent with the coordinated regulation of fatty acid metabolism. Plasma fatty acid profiles are influenced by interconnected processes including fatty acid synthesis, elongation, desaturation, transport, membrane remodeling, and lipid turnover, such that alterations in individual fatty acid species are unlikely to occur independently of the broader metabolic context [28,29]. Contemporary lipidomics studies likewise recognize that biologically relevant information may be conveyed not only by the abundance of individual lipid species but also by their covariance structure and coordinated metabolic organization [13,30,31]. Accordingly, the observed correlations should not be interpreted as evidence of direct biological interactions between individual fatty acid species but rather as statistical relationships within the measured dataset. Because the present analyses were based on a targeted panel of nine validated plasma fatty acid species, these findings are most appropriately interpreted as changes in the multivariate organization of the measured fatty acid dataset rather than comprehensive remodeling of the plasma lipidome.

### 4.3. Multivariate Organization of Behavioral Responses Following AlCl_3_ Exposure

The behavioral findings demonstrated that chronic AlCl_3_ exposure was associated with changes in the multivariate organization of the measured behavioral variables. Although spontaneous alternation identified significant behavioral impairment at day 21, the correlation and chemometric analyses indicated that the behavioral response extended beyond this individual endpoint. Spontaneous alternation showed significant between-group differences at day 21, while descriptive differences remained evident at day 30; however, inference at the later time point was limited by reduced behavioral eligibility and the presence of only one valid animal in the highest-dose group.

Importantly, despite differences in covariance patterns among the experimental groups, the behavioral dataset remained significantly organized throughout the study rather than becoming randomly structured. This interpretation was supported by the complementary chemometric analyses, in which the proportion of behavioral variance explained by the first principal component increased from 41.3% at baseline to 47.7% at day 30, while permutation analyses confirmed significant covariance organization at all time points. Together, these findings suggest that chronic AlCl_3_ exposure was associated with changes in the relationships among the measured behavioral variables in addition to alterations in individual behavioral endpoints.

These observations are consistent with contemporary behavioral neuroscience, which increasingly recognizes that complex behavioral phenotypes emerge from coordinated interactions among multiple components of the behavioral repertoire rather than from isolated behavioral variables considered independently [32,33]. Nevertheless, given the exploratory nature of the multivariate analyses, the relatively modest sample size, and the absence of complete separation among experimental groups, the present findings should be interpreted as evidence of altered statistical organization within the measured behavioral dataset rather than discrete behavioral-state transitions or specific neurobiological mechanisms.

### 4.4. Integration of Plasma Fatty Acid, Behavioral, and Biochemical Findings

The integrated matrices displayed group-specific descriptive relationships between plasma fatty acid and behavioral variables. However, no individual lipid–behavior association remained significant after FDR correction, and the integrated analyses therefore do not support specific relationships between individual fatty acid species and behavioral measures. These matrices are consequently interpreted as exploratory visualizations of cross-domain covariance rather than evidence of statistically established lipid–behavior coupling.

Brain AChE levels increased significantly only in the highest-dose group, whereas the lower exposure groups did not differ significantly from the control group. Considered alongside the plasma fatty acid and behavioral findings, this result indicates that chronic AlCl_3_ exposure was associated with concurrent changes across the measured domains. Nevertheless, the targeted fatty acid panel and limited biochemical assessment do not permit comprehensive characterization of the underlying neurotoxic mechanisms.

Collectively, the findings support the use of multivariate analyses as complementary exploratory tools for characterizing relationships among measured variables. At the same time, the absence of statistically significant lipid–behavior associations after multiple-testing correction emphasizes that cross-domain findings should be interpreted cautiously and require validation in larger cohorts with broader lipidomic and molecular coverage.

## 5. Limitations and Future Directions

The present study should be interpreted within the scope of its experimental design. Although complementary chemometric approaches, including principal component analysis, supervised modeling, correlation-based analyses, and permutation testing, consistently identified structured multivariate organizational patterns, the relatively modest sample size warrants cautious interpretation of these findings. Consequently, the observed organizational patterns should be regarded as exploratory and would benefit from confirmation in larger independent experimental cohorts.

In addition, the analytical platform employed targeted fatty acid methyl ester (FAME) profiling rather than comprehensive lipidomics. Accordingly, the present findings describe multivariate changes in the measured fatty acid profiles rather than in the complete plasma lipidome. Integration of comprehensive lipidomics with complementary transcriptomic, proteomic, and metabolomic approaches may further extend the biological interpretation of the organizational patterns identified in the present study.

Baseline multivariate heterogeneity was also observed among the experimental groups despite random allocation of animals and extensive efforts to minimize biological variability by standardizing strain, sex, age, housing conditions, and microbiome harmonization. Although such variability is inherent to biological systems, particularly in relatively small experimental cohorts, it may have contributed to the observed multivariate differences and should therefore be taken into consideration when interpreting treatment-associated organizational changes.

Furthermore, while the compositional nature of the fatty acid data was appropriately addressed through centered log-ratio (CLR) transformation and all correlation analyses were performed using CLR-transformed values with false discovery rate correction, the present work focused primarily on overall multivariate covariance organization rather than individual lipid–behavior associations. Consequently, biological interpretation was intentionally based on global organizational patterns instead of isolated pairwise relationships.

Finally, AlCl_3_ exposure was administered through drinking water, with drinking-water consumption monitored on a per-cage basis and body weight monitored throughout the study to verify consistency of the intended dosing regimen. Given the minimal between-group variation in water consumption and comparable body weights, exposure was considered consistent across groups. Nevertheless, future studies incorporating individual water-intake measurements may provide even more precise estimation of inter-animal exposure.

Overall, the present study provides an exploratory systems-level framework for investigating neurotoxicity through multivariate biological organization. Future studies incorporating larger cohorts, broader molecular profiling, complementary functional validation, and extended longitudinal designs will further refine the biological interpretation and broader applicability of the organizational framework presented here.

## 6. Conclusions

Experimental neurotoxicity studies have traditionally focused on individual behavioral or biochemical endpoints, providing limited insight into how biological systems are organized under toxicological stress. In contrast, the present study adopted an integrative systems-level framework combining spontaneous behavioral profiling, targeted plasma fatty acid methyl ester (FAME) profiling, chemometric analyses, and permutation-based statistical validation to investigate multivariate organizational patterns associated with AlCl_3_-induced neurotoxicity.

The present findings indicate that both the measured fatty acid profiles and behavioral measures exhibited significant non-random multivariate organization and that AlCl_3_ exposure was associated with progressive dose-related changes in these organizational patterns. Importantly, neurotoxic effects were characterized not simply by isolated changes in individual variables but also by changes in the covariance architecture of the measured fatty acid profiles, behavioral measures, and cholinergic function. The observed increase in alignment along dominant organizational axes further suggests progressively more structured organization of the measured variables rather than random disruption.

Collectively, these findings support the exploratory concept that pathological responses may be more informatively understood in terms of changes in multivariate biological organization rather than isolated molecular abnormalities. Although confirmation in larger independent cohorts and broader molecular profiling remains warranted, the present study demonstrates the potential value of multivariate organizational analysis as a complementary framework for investigating neurotoxic responses from a systems-level perspective.

## Figures and Tables

**Table 1:** Permutation-based validation of fatty acid profiling covariance structure at baseline and day 30.

Time Point	Observed MAC	P_MAC	Observed PC1 Variance	P_PC1
Day 0	0.233	<0.0001	0.275	0.210
Day 30	0.211	<0.001	0.320	0.023

**Table 2:** Permutation-based validation of behavioral covariance structure across time points.

Time Point	Observed MAC	P_MAC	Observed PC1 Variance	P_PC1
Day 0	0.262	0.002	0.413	0.002
Day 21	0.313	<0.0001	0.459	<0.0001
Day 30	0.336	<0.0001	0.477	<0.0001

## Data Availability

The data supporting the findings of this study are available within the article and its Supplementary Materials.

## Supplementary Materials

The following supporting information can be downloaded at https://www.mdpi.com/article/10.3390/biology15141162/s1: Table S1: Raw behavioral measurements for individual animals in the control and 50, 150, and 300 mg/kg/day AlCl_3_ groups on day 21 (A–D, respectively); Table S2: Raw behavioral measurements for individual animals in the control and 50, 150, and 300 mg/kg/day AlCl_3_ groups on day 30 (A–D, respectively); Table S3: Pearson correlation coefficients and FDR-adjusted q-values for plasma lipid variables in the control and 50, 150, and 300 mg/kg/day AlCl_3_ groups on day 30 (A–D, respectively); Table S4: Cross-validation and permutation validation of the exploratory PLS-DA models; Table S5: Pearson correlation coefficients and FDR-adjusted q-values for behavioral variables in the control and 50, 150, and 300 mg/kg/day AlCl_3_ groups on day 21 (A–D, respectively); Table S6: Pearson correlation coefficients and FDR-adjusted q-values for behavioral variables in the control and 50, 150, and 300 mg/kg/day AlCl_3_ groups on day 30 (A–D, respectively); Table S7: Pearson correlation coefficients and FDR-adjusted q-values for lipid–behavior relationships in the control and 50, 150, and 300 mg/kg/day AlCl_3_ groups on day 30 (A–D, respectively).

## Abbreviations

The following abbreviations are used in this manuscript:

AChE	Acetylcholinesterase
AlCl_3_	Aluminum Chloride
ANOVA	Analysis of Variance
BF_3_	Boron Trifluoride
CLR	Centered Log-Ratio
EI	Electron Ionization
ELISA	Enzyme-Linked Immunosorbent Assay
FAME	Fatty Acid Methyl Ester
FAME-37	37-Component Fatty Acid Methyl Ester Reference Mixture
FELASA	Federation of European Laboratory Animal Science Associations
FDR	False Discovery Rate
GC–MS	Gas Chromatography–Mass Spectrometry
HPLC	High-Performance Liquid Chromatography
IACUC	Institutional Animal Care and Use Committee
KOH	Potassium Hydroxide
MAC	Mean Absolute Correlation
MS	Mass Spectrometry
NIST	National Institute of Standards and Technology
PC	Principal Component
PC1	Principal Component 1
PC2	Principal Component 2
PCA	Principal Component Analysis
PLS-DA	Partial Least Squares–Discriminant Analysis
PUFA	Polyunsaturated Fatty Acid
SD	Standard Deviation
UPPC	University of Petra Pharmaceutical Center
