# Supplementary material for: Alterations in the Multivariate Organization of Plasma Fatty Acid Profiles and Spontaneous Behavior in an AlCl3-Induced Rat Model of Neurotoxicity
Source: Biology (Basel). 2026 Jul 16;15(14):1162. doi: 10.3390/biology15141162 (PMC13403430; doi:10.3390/biology15141162)
Supplement: Supplementary file 1 [file biology-15-01162-s001.zip › biology-4436446-supplementary.pdf]

biology MDPI

Table S1A. Raw behavioral measurements for individual animals in the control group at day 21.

Rat_ID	Lat_First_Groom (s)	Total_Arm_Entries	Rearing_Count	Grooming_Count	Head_Dip_Count
Rat1	60	17	11	5	6
Rat2	120	7	4	5	10
Rat3	20	9	2	7	10
Rat4	90	9	12	5	14
Rat5	90	6	1	5	9
Rat6	90	9	13	3	12
Rat7	80	5	6	3	5
Rat8	40	3	12	3	5

Table S1B. Raw behavioral measurements for individual animals in the 50 mg/kg/day $AlCl_3$ group at day 21.

Rat_ID	Lat_First_Groom (s)	Total_Arm_Entries	Rearing_Count	Grooming_Count	Head_Dip_Count
Rat1	150	4	1	1	1
Rat2	30	5	15	3	6
Rat3	300	7	1	0	4
Rat4	60	6	0	2	6
Rat5	80	15	5	3	13
Rat6	120	1	4	1	12
Rat7	60	4	9	4	8
Rat8	100	4	0	2	1

Table S1C. Raw behavioral measurements for individual animals in the 150 mg/kg/day $AlCl_3$ group at day 21.

Rat_ID	Lat_First_Groom (s)	Total_Arm_Entries	Rearing_Count	Grooming_Count	Head_Dip_Count
Rat1	60	3	2	1	8
Rat2	60	13	9	6	12
Rat3	140	7	0	2	7
Rat4	45	6	6	5	10
Rat5	195	16	5	2	10
Rat6	30	4	10	3	4
Rat7	120	9	1	2	2
Rat8	40	8	9	3	13

Table S1D. Raw behavioral measurements for individual animals in the 300 mg/kg/day $AlCl_3$ group at day 21.

Rat_ID	Lat_First_Groom (s)	Total_Arm_Entries	Rearing_Count	Grooming_Count	Head_Dip_Count
Rat1	130	9	3	1	5
Rat2	165	2	0	1	6
Rat3	20	3	1	2	8
Rat4	180	3	0	1	2
Rat5	40	7	0	2	1
Rat6	95	8	6	2	6
Rat7	240	3	2	1	2
Rat8	300	8	15	0	12

Table S2A. Raw behavioral measurements for individual animals in the control group at day 30.

Rat_ID	Lat_First_Groom (s)	Total_Arm_Entries	Rearing_Count	Grooming_Count	Head_Dip_Count
Rat1	105	13	5	3	4
Rat2	45	5	2	2	6
Rat3	20	10	0	10	0
Rat4	70	6	3	3	5
Rat5	80	4	1	2	5
Rat6	65	10	9	5	17
Rat7	20	1	4	3	4
Rat8	55	3	6	2	5

Table S2B. Raw behavioral measurements for individual animals in the 50 mg/kg/day $AlCl_3$ group at day 30.

Rat_ID	Lat_First_Groom (s)	Total_Arm_Entries	Rearing_Count	Grooming_Count	Head_Dip_Count
Rat1	300	2	2	0	1
Rat2	50	12	4	2	4
Rat3	300	2	4	0	2
Rat4	25	7	3	5	6
Rat5	300	14	1	0	6
Rat6	45	4	4	5	6
Rat7	35	2	5	2	1
Rat8	15	4	0	3	1

Table S2C. Raw behavioral measurements for individual animals in the 150 mg/kg/day $AlCl_3$ group at day 30.

Rat_ID	Lat_First_Groom (s)	Total_Arm_Entries	Rearing_Count	Grooming_Count	Head_Dip_Count
Rat1	35	2	1	2	1
Rat2	40	4	0	2	2
Rat3	45	5	0	1	0
Rat4	300	8	2	0	1
Rat5	300	7	1	0	3
Rat6	45	1	0	3	2
Rat7	75	5	3	2	4
Rat8	45	6	5	3	7

Table S2D. Raw behavioral measurements for individual animals in the 300 mg/kg/day $AlCl_3$ group at day 30.

Rat_ID	Lat_First_Groom (s)	Total_Arm_Entries	Rearing_Count	Grooming_Count	Head_Dip_Count
Rat1	300	4	1	0	1
Rat2	235	3	0	1	1
Rat3	165	5	1	2	5
Rat4	40	2	1	3	3
Rat5	300	10	0	0	0
Rat6	300	3	0	0	1
Rat7	300	1	2	0	1
Rat8	300	3	1	0	0

Table S3A. Pearson correlation coefficients (r) and FDR-adjusted q-values for plasma lipid variables at day 30 in the control group.

Variable	Lipid 1	Lipid 2	Lipid 3	Lipid 4	Lipid 5	Lipid 6	Lipid 7	Lipid 8	Lipid 9
Lipid 1	—	-0.53 (0.7313)	0.21 (0.9947)	0.29 (0.9947)	-0.22 (0.9947)	0.05 (0.9947)	-0.53 (0.7313)	0.08 (0.9947)	-0.05 (0.9947)
Lipid 2	-0.53 (0.7313)	—	-0.77 (0.3802)	-0.30 (0.9947)	0.14 (0.9947)	0.02 (0.9947)	0.21 (0.9947)	-0.26 (0.9947)	-0.12 (0.9947)
Lipid 3	0.21 (0.9947)	-0.77 (0.3802)	—	-0.03 (0.9947)	0.37 (0.9335)	-0.16 (0.9947)	0.38 (0.9335)	-0.13 (0.9947)	-0.20 (0.9947)
Lipid 4	0.29 (0.9947)	-0.30 (0.9947)	-0.03 (0.9947)	—	-0.82 (0.3802)	-0.59 (0.7313)	-0.02 (0.9947)	-0.38 (0.9335)	-0.43 (0.9335)
Lipid 5	-0.22 (0.9947)	0.14 (0.9947)	0.37 (0.9335)	-0.82 (0.3802)	—	0.15 (0.9947)	0.38 (0.9335)	-0.00 (0.9969)	0.03 (0.9947)
Lipid 6	0.05 (0.9947)	0.02 (0.9947)	-0.16 (0.9947)	-0.59 (0.7313)	0.15 (0.9947)	—	-0.53 (0.7313)	0.65 (0.6929)	0.56 (0.7313)
Lipid 7	-0.53 (0.7313)	0.21 (0.9947)	0.38 (0.9335)	-0.02 (0.9947)	0.38 (0.9335)	-0.53 (0.7313)	—	-0.77 (0.3802)	-0.69 (0.6216)
Lipid 8	0.08 (0.9947)	-0.26 (0.9947)	-0.13 (0.9947)	-0.38 (0.9335)	-0.00 (0.9969)	0.65 (0.6929)	-0.77 (0.3802)	—	0.97 (0.008189)
Lipid 9	-0.05 (0.9947)	-0.12 (0.9947)	-0.20 (0.9947)	-0.43 (0.9335)	0.03 (0.9947)	0.56 (0.7313)	-0.69 (0.6216)	0.97 (0.008189)	—

https://doi.org/10.3390/biology15141162

Table S3B. Pearson correlation coefficients (r) and FDR-adjusted q-values for plasma lipid variables at day 30 in the 50 mg/kg/day $AlCl_3$ group.

Variable	Lipid 1	Lipid 2	Lipid 3	Lipid 4	Lipid 5	Lipid 6	Lipid 7	Lipid 8	Lipid 9
Lipid 1	—	-0.45 (0.9331)	0.60 (0.9331)	-0.27 (0.9331)	-0.66 (0.9331)	-0.32 (0.9331)	-0.36 (0.9331)	-0.46 (0.9331)	-0.18 (0.9819)
Lipid 2	-0.45 (0.9331)	—	0.08 (0.9843)	-0.26 (0.9331)	-0.05 (0.9843)	-0.03 (0.9843)	-0.10 (0.9843)	-0.08 (0.9843)	-0.01 (0.9843)
Lipid 3	0.60 (0.9331)	0.08 (0.9843)	—	-0.33 (0.9331)	-0.44 (0.9331)	-0.35 (0.9331)	-0.28 (0.9331)	-0.65 (0.9331)	-0.58 (0.9331)
Lipid 4	-0.27 (0.9331)	-0.26 (0.9331)	-0.33 (0.9331)	—	0.02 (0.9843)	0.09 (0.9843)	-0.28 (0.9331)	0.86 (0.235)	-0.17 (0.9819)
Lipid 5	-0.66 (0.9331)	-0.05 (0.9843)	-0.44 (0.9331)	0.02 (0.9843)	—	-0.12 (0.9843)	0.50 (0.9331)	0.23 (0.9424)	0.47 (0.9331)
Lipid 6	-0.32 (0.9331)	-0.03 (0.9843)	-0.35 (0.9331)	0.09 (0.9843)	-0.12 (0.9843)	—	0.21 (0.9664)	0.01 (0.9843)	-0.36 (0.9331)
Lipid 7	-0.36 (0.9331)	-0.10 (0.9843)	-0.28 (0.9331)	-0.28 (0.9331)	0.50 (0.9331)	0.21 (0.9664)	—	-0.26 (0.9331)	0.04 (0.9843)
Lipid 8	-0.46 (0.9331)	-0.08 (0.9843)	-0.65 (0.9331)	0.86 (0.235)	0.23 (0.9424)	0.01 (0.9843)	-0.26 (0.9331)	—	0.25 (0.9331)
Lipid 9	-0.18 (0.9819)	-0.01 (0.9843)	-0.58 (0.9331)	-0.17 (0.9819)	0.47 (0.9331)	-0.36 (0.9331)	0.04 (0.9843)	0.25 (0.9331)	—

Table S3C. Pearson correlation coefficients (r) and FDR-adjusted q-values for plasma lipid variables at day 30 in the 150 mg/kg/day $AlCl_3$ group.

Variable	Lipid 1	Lipid 2	Lipid 3	Lipid 4	Lipid 5	Lipid 6	Lipid 7	Lipid 8	Lipid 9
Lipid 1	—	0.04 (0.9569)	-0.43 (0.7859)	0.29 (0.8866)	0.23 (0.8866)	-0.35 (0.7859)	0.23 (0.8866)	0.52 (0.7578)	-0.02 (0.9569)
Lipid 2	0.04 (0.9569)	—	-0.38 (0.7859)	0.72 (0.7571)	0.18 (0.8866)	-0.20 (0.8866)	-0.36 (0.7859)	0.39 (0.7859)	0.50 (0.7578)
Lipid 3	-0.43 (0.7859)	-0.38 (0.7859)	—	-0.21 (0.8866)	-0.05 (0.9569)	-0.16 (0.8866)	-0.10 (0.911)	-0.42 (0.7859)	-0.14 (0.8866)
Lipid 4	0.29 (0.8866)	0.72 (0.7571)	-0.21 (0.8866)	—	0.42 (0.7859)	-0.50 (0.7578)	0.04 (0.9569)	0.14 (0.8866)	0.26 (0.8866)
Lipid 5	0.23 (0.8866)	0.18 (0.8866)	-0.05 (0.9569)	0.42 (0.7859)	—	-0.84 (0.3124)	0.61 (0.7578)	-0.18 (0.8866)	0.59 (0.7578)
Lipid 6	-0.35 (0.7859)	-0.20 (0.8866)	-0.16 (0.8866)	-0.50 (0.7578)	-0.84 (0.3124)	—	-0.59 (0.7578)	0.13 (0.8866)	-0.65 (0.7578)
Lipid 7	0.23 (0.8866)	-0.36 (0.7859)	-0.10 (0.911)	0.04 (0.9569)	0.61 (0.7578)	-0.59 (0.7578)	—	-0.55 (0.7578)	0.36 (0.7859)
Lipid 8	0.52 (0.7578)	0.39 (0.7859)	-0.42 (0.7859)	0.14 (0.8866)	-0.18 (0.8866)	0.13 (0.8866)	-0.55 (0.7578)	—	-0.16 (0.8866)
Lipid 9	-0.02 (0.9569)	0.50 (0.7578)	-0.14 (0.8866)	0.26 (0.8866)	0.59 (0.7578)	-0.65 (0.7578)	0.36 (0.7859)	-0.16 (0.8866)	—

Table S3D. Pearson correlation coefficients (r) and FDR-adjusted q-values for plasma lipid variables at day 30 in the 300 mg/kg/day $AlCl_3$ group.

Variable	Lipid 1	Lipid 2	Lipid 3	Lipid 4	Lipid 5	Lipid 6	Lipid 7	Lipid 8	Lipid 9
Lipid 1	—	-0.55 (0.3561)	0.98 (0.001123)	-0.40 (0.5239)	-0.77 (0.1209)	-0.70 (0.1607)	-0.78 (0.1209)	-0.20 (0.864)	-0.57 (0.3503)
Lipid 2	-0.55 (0.3561)	—	-0.47 (0.4303)	0.20 (0.864)	0.19 (0.864)	-0.13 (0.8689)	0.14 (0.8689)	-0.25 (0.8323)	-0.10 (0.8689)
Lipid 3	0.98 (0.001123)	-0.47 (0.4303)	—	-0.49 (0.4067)	-0.78 (0.1209)	-0.71 (0.1607)	-0.77 (0.1209)	-0.12 (0.8689)	-0.64 (0.2333)
Lipid 4	-0.40 (0.5239)	0.20 (0.864)	-0.49 (0.4067)	—	0.14 (0.8689)	-0.01 (0.9996)	-0.10 (0.8689)	-0.42 (0.518)	0.00 (0.9996)
Lipid 5	-0.77 (0.1209)	0.19 (0.864)	-0.78 (0.1209)	0.14 (0.8689)	—	0.73 (0.1414)	0.73 (0.1414)	0.13 (0.8689)	0.53 (0.3561)
Lipid 6	-0.70 (0.1607)	-0.13 (0.8689)	-0.71 (0.1607)	-0.01 (0.9996)	0.73 (0.1414)	—	0.93 (0.01211)	0.54 (0.3561)	0.88 (0.04499)
Lipid 7	-0.78 (0.1209)	0.14 (0.8689)	-0.77 (0.1209)	-0.10 (0.8689)	0.73 (0.1414)	0.93 (0.01211)	—	0.53 (0.3561)	0.86 (0.05159)
Lipid 8	-0.20 (0.864)	-0.25 (0.8323)	-0.12 (0.8689)	-0.42 (0.518)	0.13 (0.8689)	0.54 (0.3561)	0.53 (0.3561)	—	0.36 (0.6011)
Lipid 9	-0.57 (0.3503)	-0.10 (0.8689)	-0.64 (0.2333)	0.00 (0.9996)	0.53 (0.3561)	0.88 (0.04499)	0.86 (0.05159)	0.36 (0.6011)	—

Table S4. Cross-validation and permutation validation of the exploratory PLS-DA models. Model performance is summarized by the proportion of variance explained in the predictor matrix (R^2X), the proportion of variance explained in class membership (R^2Y), the cross-validated predictive ability (Q^2), and the permutation test significance (pQ^2; 1000 permutations). Leave-one-out cross-validation was applied to the Water vs All Treated comparison, whereas 7-fold cross-validation was used for pairwise comparisons.

Comparison	Cross-validation	R^2X	R^2Y	Q^2	Permutation test (pQ^2)
Water vs All Treated	Leave-one-out	0.199	0.114	−0.142	0.473
Water vs 50 mg/kg	7-fold	0.178	0.439	−0.079	0.440
Water vs 150 mg/kg	7-fold	0.285	0.564	0.218	0.101
Water vs 300 mg/kg	7-fold	0.229	0.284	−0.216	0.606

Table S5A. Pearson correlation coefficients (r) and FDR-adjusted q-values for behavioral variables at day 21 in the control group.

Variable	Lat_First_Groom_s	Total_Arm_Entries	Rearing_Count	Grooming_Count	Head_Dip_Count
Lat_First_Groom_s	—	-0.08 (0.9682)	-0.02 (0.9682)	-0.27 (0.9507)	0.37 (0.9507)
Total_Arm_Entries	-0.08 (0.9682)	—	0.24 (0.9507)	0.40 (0.9507)	0.14 (0.9682)
Rearing_Count	-0.02 (0.9682)	0.24 (0.9507)	—	-0.56 (0.9507)	0.07 (0.9682)
Grooming_Count	-0.27 (0.9507)	0.40 (0.9507)	-0.56 (0.9507)	—	0.35 (0.9507)
Head_Dip_Count	0.37 (0.9507)	0.14 (0.9682)	0.07 (0.9682)	0.35 (0.9507)	—

Table S5B. Pearson correlation coefficients (r) and FDR-adjusted q-values for behavioral variables at day 21 in the 50 mg/kg/day $AlCl_3$ group.

Variable	Lat_First_Groom_s	Total_Arm_Entries	Rearing_Count	Grooming_Count	Head_Dip_Count
Lat_First_Groom_s	—	-0.00 (0.9969)	-0.52 (0.6348)	-0.82 (0.117)	-0.30 (0.6825)
Total_Arm_Entries	-0.00 (0.9969)	—	-0.00 (0.9969)	0.24 (0.7136)	0.37 (0.6825)
Rearing_Count	-0.52 (0.6348)	-0.00 (0.9969)	—	0.64 (0.439)	0.34 (0.6825)
Grooming_Count	-0.82 (0.117)	0.24 (0.7136)	0.64 (0.439)	—	0.34 (0.6825)
Head_Dip_Count	-0.30 (0.6825)	0.37 (0.6825)	0.34 (0.6825)	0.34 (0.6825)	—

Table S5C. Pearson correlation coefficients (r) and FDR-adjusted q-values for behavioral variables at day 21 in the 150 mg/kg/day $AlCl_3$ group.

Variable	Lat_First_Groom_s	Total_Arm_Entries	Rearing_Count	Grooming_Count	Head_Dip_Count
Lat_First_Groom_s	—	0.64 (0.4371)	-0.58 (0.4371)	-0.44 (0.4628)	-0.15 (0.792)
Total_Arm_Entries	0.64 (0.4371)	—	0.11 (0.792)	0.25 (0.6886)	0.36 (0.5395)
Rearing_Count	-0.58 (0.4371)	0.11 (0.792)	—	0.63 (0.4371)	0.45 (0.4628)
Grooming_Count	-0.44 (0.4628)	0.25 (0.6886)	0.63 (0.4371)	—	0.47 (0.4628)
Head_Dip_Count	-0.15 (0.792)	0.36 (0.5395)	0.45 (0.4628)	0.47 (0.4628)	—

Table S5D. Pearson correlation coefficients (r) and FDR-adjusted q-values for behavioral variables at day 21 in the 300 mg/kg/day $AlCl_3$ group.

Variable	Lat_First_Groom_s	Total_Arm_Entries	Rearing_Count	Grooming_Count	Head_Dip_Count
Lat_First_Groom_s	—	-0.01 (0.972)	0.59 (0.2791)	-0.92 (0.01031)	0.29 (0.692)
Total_Arm_Entries	-0.01 (0.972)	—	0.57 (0.2791)	-0.05 (0.972)	0.25 (0.692)
Rearing_Count	0.59 (0.2791)	0.57 (0.2791)	—	-0.58 (0.2791)	0.77 (0.1237)
Grooming_Count	-0.92 (0.01031)	-0.05 (0.972)	-0.58 (0.2791)	—	-0.41 (0.5119)
Head_Dip_Count	0.29 (0.692)	0.25 (0.692)	0.77 (0.1237)	-0.41 (0.5119)	—

Table S6A. Pearson correlation coefficients (r) and FDR-adjusted q-values for behavioral variables at day 30 in the control group.

Variable	Lat_First_Groom_s	Total_Arm_Entries	Rearing_Count	Grooming_Count	Head_Dip_Count
Lat_First_Groom_s	—	0.05 (0.9225)	0.24 (0.8398)	-0.52 (0.716)	0.47 (0.716)
Total_Arm_Entries	0.05 (0.9225)	—	0.07 (0.9225)	0.75 (0.2645)	0.33 (0.8398)
Rearing_Count	0.24 (0.8398)	0.07 (0.9225)	—	-0.23 (0.8398)	0.83 (0.2009)
Grooming_Count	-0.52 (0.716)	0.75 (0.2645)	-0.23 (0.8398)	—	-0.20 (0.8398)
Head_Dip_Count	0.47 (0.716)	0.33 (0.8398)	0.83 (0.2009)	-0.20 (0.8398)	—

Table S6B. Pearson correlation coefficients (r) and FDR-adjusted q-values for behavioral variables at day 30 in the 50 mg/kg/day $AlCl_3$ group.

Variable	Lat_First_Groom_s	Total_Arm_Entries	Rearing_Count	Grooming_Count	Head_Dip_Count
Lat_First_Groom_s	—	0.04 (0.9187)	-0.21 (0.9187)	-0.84 (0.08778)	-0.10 (0.9187)
Total_Arm_Entries	0.04 (0.9187)	—	-0.25 (0.9187)	-0.07 (0.9187)	0.65 (0.4065)
Rearing_Count	-0.21 (0.9187)	-0.25 (0.9187)	—	0.16 (0.9187)	0.05 (0.9187)
Grooming_Count	-0.84 (0.08778)	-0.07 (0.9187)	0.16 (0.9187)	—	0.45 (0.8962)
Head_Dip_Count	-0.10 (0.9187)	0.65 (0.4065)	0.05 (0.9187)	0.45 (0.8962)	—

Table S6C. Pearson correlation coefficients (r) and FDR-adjusted q-values for behavioral variables at day 30 in the 150 mg/kg/day $AlCl_3$ group.

Variable	Lat_First_Groom_s	Total_Arm_Entries	Rearing_Count	Grooming_Count	Head_Dip_Count
Lat_First_Groom_s	—	0.74 (0.1223)	0.04 (0.9261)	-0.84 (0.04447)	-0.11 (0.8915)
Total_Arm_Entries	0.74 (0.1223)	—	0.44 (0.4568)	-0.70 (0.1377)	0.22 (0.7544)
Rearing_Count	0.04 (0.9261)	0.44 (0.4568)	—	0.24 (0.7544)	0.84 (0.04447)
Grooming_Count	-0.84 (0.04447)	-0.70 (0.1377)	0.24 (0.7544)	—	0.46 (0.4568)
Head_Dip_Count	-0.11 (0.8915)	0.22 (0.7544)	0.84 (0.04447)	0.46 (0.4568)	—

Table S6D. Pearson correlation coefficients (r) and FDR-adjusted q-values for behavioral variables at day 30 in the 300 mg/kg/day $AlCl_3$ group.

Variable	Lat_First_Groom_s	Total_Arm_Entries	Rearing_Count	Grooming_Count	Head_Dip_Count
Lat_First_Groom_s	—	0.21 (0.8382)	-0.11 (0.8382)	-0.99 (2.059e-05)	-0.74 (0.1247)
Total_Arm_Entries	0.21 (0.8382)	—	-0.53 (0.4346)	-0.19 (0.8382)	-0.14 (0.8382)
Rearing_Count	-0.11 (0.8382)	-0.53 (0.4346)	—	0.09 (0.8382)	0.24 (0.8382)
Grooming_Count	-0.99 (2.059e-05)	-0.19 (0.8382)	0.09 (0.8382)	—	0.80 (0.08802)
Head_Dip_Count	-0.74 (0.1247)	-0.14 (0.8382)	0.24 (0.8382)	0.80 (0.08802)	—

https://doi.org/10.3390/biology15141162

Table S7A. Pearson correlation coefficients (r) and FDR-adjusted q-values for lipid–behavior relationships at day 30 in the control group.

Lipid	Latency	Arm	Rearing	Groom	Head
Lipid 1	0.18 (0.9745)	0.55 (0.9688)	0.68 (0.8257)	0.09 (0.9807)	0.91 (0.2282)
Lipid 2	-0.11 (0.9745)	0.12 (0.9745)	-0.40 (0.9745)	0.38 (0.9745)	-0.58 (0.9688)
Lipid 3	-0.24 (0.9745)	-0.26 (0.9745)	0.45 (0.9745)	-0.16 (0.9745)	0.31 (0.9745)
Lipid 4	0.86 (0.4072)	0.25 (0.9745)	0.15 (0.9745)	-0.23 (0.9745)	0.52 (0.9688)
Lipid 5	-0.66 (0.8757)	-0.31 (0.9745)	0.25 (0.9745)	0.10 (0.9807)	-0.28 (0.9745)
Lipid 6	-0.56 (0.9688)	-0.27 (0.9745)	-0.22 (0.9745)	-0.03 (0.9888)	-0.19 (0.9745)
Lipid 7	-0.15 (0.9745)	-0.22 (0.9745)	0.00 (0.9969)	0.18 (0.9745)	-0.32 (0.9745)
Lipid 8	-0.25 (0.9745)	-0.16 (0.9745)	-0.31 (0.9745)	-0.26 (0.9745)	-0.15 (0.9745)
Lipid 9	-0.30 (0.9745)	-0.09 (0.9807)	-0.45 (0.9745)	-0.12 (0.9745)	-0.31 (0.9745)

Table S7B. Pearson correlation coefficients (r) and FDR-adjusted q-values for lipid–behavior relationships at day 30 in the 50 mg/kg/day $AlCl_3$ group.

Lipid	Latency	Arm	Rearing	Groom	Head
Lipid 1	-0.12 (0.9843)	-0.30 (0.9396)	0.41 (0.9396)	0.42 (0.9396)	0.33 (0.9396)
Lipid 2	-0.05 (0.9843)	-0.22 (0.9396)	-0.68 (0.7496)	-0.20 (0.9396)	-0.62 (0.7496)
Lipid 3	-0.58 (0.7521)	-0.18 (0.9402)	-0.13 (0.9843)	0.84 (0.2166)	0.36 (0.9396)
Lipid 4	0.35 (0.9396)	0.92 (0.1201)	-0.22 (0.9396)	-0.30 (0.9396)	0.63 (0.7496)
Lipid 5	-0.12 (0.9843)	-0.02 (0.9843)	0.23 (0.9396)	-0.04 (0.9843)	-0.15 (0.9572)
Lipid 6	0.32 (0.9396)	0.20 (0.9396)	-0.38 (0.9396)	-0.29 (0.9396)	-0.09 (0.9843)
Lipid 7	-0.56 (0.7816)	-0.04 (0.9843)	0.38 (0.9396)	0.20 (0.9396)	-0.30 (0.9396)
Lipid 8	0.59 (0.7521)	0.64 (0.7496)	-0.19 (0.9402)	-0.66 (0.7496)	0.22 (0.9396)
Lipid 9	0.39 (0.9396)	-0.41 (0.9396)	0.50 (0.9089)	-0.55 (0.7838)	-0.51 (0.9089)

Table S7C. Pearson correlation coefficients (r) and FDR-adjusted q-values for lipid–behavior relationships at day 30 in the 150 mg/kg/day $AlCl_3$ group.

Lipid	Latency	Arm	Rearing	Groom	Head
Lipid 1	-0.12 (0.9701)	-0.11 (0.9701)	0.21 (0.9451)	0.33 (0.9451)	0.48 (0.9451)
Lipid 2	-0.02 (0.9929)	-0.35 (0.9451)	0.24 (0.9451)	0.24 (0.9451)	0.21 (0.9451)
Lipid 3	-0.29 (0.9451)	-0.28 (0.9451)	-0.50 (0.9451)	0.11 (0.9701)	-0.24 (0.9451)
Lipid 4	0.24 (0.9451)	0.03 (0.9929)	0.28 (0.9451)	-0.00 (0.9993)	0.48 (0.9451)
Lipid 5	-0.22 (0.9451)	0.02 (0.9929)	0.33 (0.9451)	0.36 (0.9451)	0.58 (0.9451)
Lipid 6	0.34 (0.9451)	0.30 (0.9451)	0.10 (0.9701)	-0.36 (0.9451)	-0.31 (0.9451)
Lipid 7	-0.01 (0.9929)	0.36 (0.9451)	0.02 (0.9929)	-0.21 (0.9451)	0.11 (0.9701)
Lipid 8	0.04 (0.9929)	-0.39 (0.9451)	0.02 (0.9929)	0.35 (0.9451)	0.13 (0.9701)
Lipid 9	-0.54 (0.9451)	-0.47 (0.9451)	-0.02 (0.9929)	0.40 (0.9451)	0.01 (0.9929)

Table S7D. Pearson correlation coefficients (r) and FDR-adjusted q-values for lipid–behavior relationships at day 30 in the 300 mg/kg/day $AlCl_3$ group.

Lipid	Latency	Arm	Rearing	Groom	Head
Lipid 1	0.11 (0.9644)	0.61 (0.4714)	-0.72 (0.2771)	-0.05 (0.9644)	-0.20 (0.9583)
Lipid 2	-0.64 (0.3952)	-0.56 (0.4977)	0.56 (0.4977)	0.56 (0.4977)	0.28 (0.9039)
Lipid 3	0.17 (0.9644)	0.56 (0.4977)	-0.69 (0.2939)	-0.13 (0.9644)	-0.37 (0.7938)
Lipid 4	-0.02 (0.9846)	-0.31 (0.8907)	0.74 (0.2554)	0.08 (0.9644)	0.50 (0.5581)
Lipid 5	0.07 (0.9644)	-0.39 (0.7719)	0.20 (0.9583)	-0.10 (0.9644)	0.17 (0.9644)
Lipid 6	0.28 (0.9039)	-0.30 (0.8907)	0.22 (0.9583)	-0.30 (0.8907)	-0.08 (0.9644)
Lipid 7	-0.02 (0.9846)	-0.33 (0.8733)	0.28 (0.9039)	-0.03 (0.9846)	0.06 (0.9644)
Lipid 8	0.46 (0.6139)	0.38 (0.7740)	-0.05 (0.9644)	-0.53 (0.4977)	-0.58 (0.4977)
Lipid 9	-0.05 (0.9644)	-0.30 (0.8907)	0.14 (0.9644)	0.06 (0.9644)	0.21 (0.9583)

Disclaimer/Publisher's Note: The statements, opinions and data contained in all publications are solely those of the individual author(s) and contributor(s) and not of MDPI and/or the editor(s). MDPI and/or the editor(s) disclaim responsibility for any injury to people or property resulting from any ideas, methods, instructions or products referred to in the content.
